# Supplementary material for: Time-series transcriptome analysis identified differentially expressed genes in broiler chicken infected with mixed Eimeria species
Source: Front Genet. 2022 Aug 8;13:886781. doi: 10.3389/fgene.2022.886781 (PMC9393255; doi:10.3389/fgene.2022.886781)
Supplement: Supplementary file 2 [file DataSheet1.ZIP › 4dpi_KEGG.Gsea.1625100298584/KEGG_RIG_I_LIKE_RECEPTOR_SIGNALING_PATHWAY.html]

Details for gene set KEGG\_RIG\_I\_LIKE\_RECEPTOR\_SIGNALING\_PATHWAY[GSEA]

|  || Dataset | TMM\_4dpi\_gct\_format\_4dpi\_gct\_format.Class\_4dpi.cls #PC\_versus\_NC.Class\_4dpi.cls #PC\_versus\_NC\_repos |
| Phenotype | Class\_4dpi.cls#PC\_versus\_NC\_repos |
| Upregulated in class | 1 |
| GeneSet | KEGG\_RIG\_I\_LIKE\_RECEPTOR\_SIGNALING\_PATHWAY |
| Enrichment Score (ES) | 0.5524085 |
| Normalized Enrichment Score (NES) | 1.8278751 |
| Nominal p-value | 0.0017349063 |
| FDR q-value | 0.03645708 |
| FWER p-Value | 0.1332 |
Table: GSEA Results Summary

  

Fig 1: Enrichment plot: KEGG\_RIG\_I\_LIKE\_RECEPTOR\_SIGNALING\_PATHWAY      
 Profile of the Running ES Score & Positions of GeneSet Members on the Rank Ordered List

  

| SYMBOL | TITLE | RANK IN GENE LIST | RANK METRIC SCORE | RUNNING ES | CORE ENRICHMENT || 1 | IFIH1 | na | 23 | 2.222 | 0.1429 | Yes |
| 2 | IRF7 | na | 85 | 1.706 | 0.2491 | Yes |
| 3 | DHX58 | na | 116 | 1.564 | 0.3485 | Yes |
| 4 | TRIM25 | na | 408 | 1.056 | 0.3931 | Yes |
| 5 | IKBKE | na | 505 | 0.959 | 0.4476 | Yes |
| 6 | TRADD | na | 711 | 0.810 | 0.4833 | Yes |
| 7 | MAPK13 | na | 870 | 0.727 | 0.5175 | Yes |
| 8 | TANK | na | 982 | 0.678 | 0.5524 | Yes |
| 9 | MAPK12 | na | 2084 | 0.408 | 0.4870 | No |
| 10 | CASP8 | na | 2138 | 0.398 | 0.5085 | No |
| 11 | MAPK9 | na | 2786 | 0.310 | 0.4747 | No |
| 12 | FADD | na | 2797 | 0.309 | 0.4940 | No |
| 13 | AZI2 | na | 2840 | 0.304 | 0.5103 | No |
| 14 | NLRX1 | na | 3156 | 0.262 | 0.5011 | No |
| 15 | IKBKB | na | 3506 | 0.220 | 0.4863 | No |
| 16 | TKFC | na | 3694 | 0.199 | 0.4836 | No |
| 17 | TRAF6 | na | 3778 | 0.190 | 0.4890 | No |
| 18 | MAPK14 | na | 4107 | 0.159 | 0.4720 | No |
| 19 | RIPK1 | na | 4121 | 0.158 | 0.4812 | No |
| 20 | TRAF2 | na | 4731 | 0.103 | 0.4371 | No |
| 21 | TBK1 | na | 4986 | 0.079 | 0.4210 | No |
| 22 | ATG5 | na | 5100 | 0.070 | 0.4162 | No |
| 23 | TRAF3 | na | 5463 | 0.040 | 0.3885 | No |
| 24 | NFKBIA | na | 5648 | 0.021 | 0.3745 | No |
| 25 | MAPK10 | na | 5730 | 0.014 | 0.3687 | No |
| 26 | RELA | na | 5979 | -0.005 | 0.3483 | No |
| 27 | SIKE1 | na | 6142 | -0.018 | 0.3359 | No |
| 28 | ATG12 | na | 6158 | -0.019 | 0.3359 | No |
| 29 | MAVS | na | 6400 | -0.038 | 0.3183 | No |
| 30 | MAP3K7 | na | 6443 | -0.042 | 0.3175 | No |
| 31 | NFKB1 | na | 6765 | -0.068 | 0.2951 | No |
| 32 | MAPK8 | na | 6831 | -0.073 | 0.2944 | No |
| 33 | DDX3X | na | 6973 | -0.087 | 0.2883 | No |
| 34 | MAP3K1 | na | 8024 | -0.182 | 0.2124 | No |
| 35 | NFKBIB | na | 8760 | -0.257 | 0.1678 | No |
| 36 | CHUK | na | 9400 | -0.336 | 0.1363 | No |
| 37 | CYLD | na | 9639 | -0.367 | 0.1404 | No |
| 38 | PIN1 | na | 10155 | -0.438 | 0.1259 | No |
| 39 | MAPK11 | na | 10200 | -0.444 | 0.1512 | No |
Table: GSEA details [plain text format]

  

Fig 2: KEGG\_RIG\_I\_LIKE\_RECEPTOR\_SIGNALING\_PATHWAY      
 Blue-Pink O' Gram in the Space of the Analyzed GeneSet

  

Fig 3: KEGG\_RIG\_I\_LIKE\_RECEPTOR\_SIGNALING\_PATHWAY: Random ES distribution      
 Gene set null distribution of ES for **KEGG\_RIG\_I\_LIKE\_RECEPTOR\_SIGNALING\_PATHWAY**
